# Supplementary material for: Stress-primed secretory autophagy promotes extracellular BDNF maturation by enhancing MMP9 secretion
Source: Nat Commun. 2021 Jul 30;12:4643. doi: 10.1038/s41467-021-24810-5 (PMC8324795; doi:10.1038/s41467-021-24810-5)
Supplement: Supplementary file 1 — Supplementary Figures and references [file 41467_2021_24810_MOESM1_ESM.pdf]

Supplementary Figures

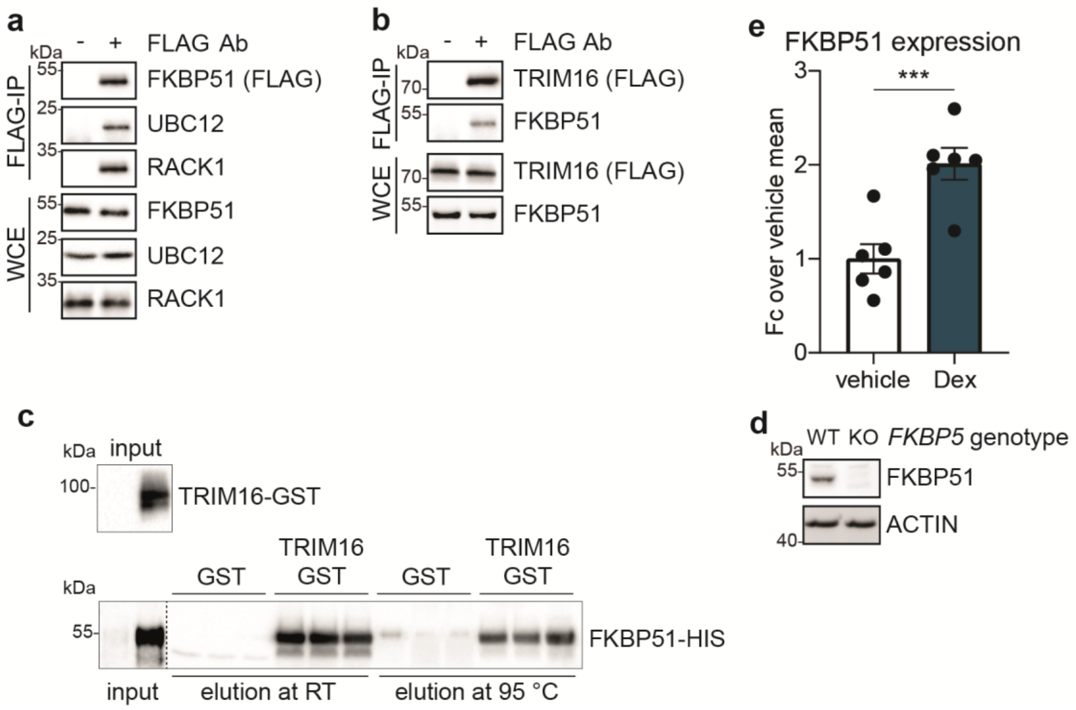

Figure S1

a) Western blotting for FKBP51, UBC12 and RACK1 in FLAG-tagged FKBP51 co-IP (FLAG-IP) and whole cell extract (WCE) as control. **b)** Western blotting for TRIM16 and FKBP51 in FLAG-tagged TRIM16 co-IP (FLAG-IP) and whole cell extract (WCE) as control. **c)** GST pull down assay. HIS-tagged FKBP51 was incubated with purified magnetic beads-GST-tagged TRIM16 or magnetic beads-GST protein alone. After incubation, bead bound proteins were eluted at room temperature (RT) or at 95 °C and subjected to western blot analysis using antibodies against GST and HIS. Input lane contains GST alone (left) or GST-tagged TRIM16 (right). The dotted line separates the blot shown into two parts according to blot exposition time. Due to the high amount of recombinant FKBP51-HIS of the input a shorter exposition time was selected for imaging. **d)** Western blotting for FKBP51 in WT and *FKBP5* KO SH-SY5Y cells. **e)** Quantifications of western blots for FKBP51 in SH-SY5Y cells treated with vehicle or 100 nM dexamethasone (Dex) for 4 hours. Data shown as mean ± s.e.m. of n=6 biologically independent samples. Unpaired one-tailed t-test was performed; ***$P < 0.001$. Ab, antibody; Fc, fold change.

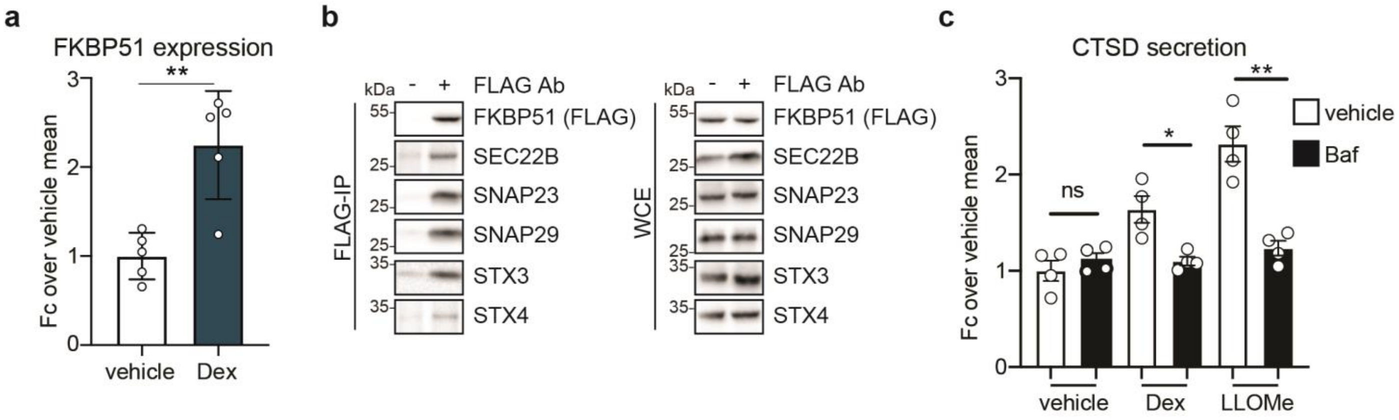

Figure S2

a) Quantifications of western blots for FKBP51 in SIMA-9 cells treated with vehicle or 300 nM dexamethasone (Dex) for 4 hours. Data shown as mean ± s.e.m. of n=5 biologically independent samples. Unpaired one-tailed t-test was performed; **p < 0.01. Fc, fold change. **b)** Western blotting for FKBP51, SEC22B, SNAP23, SNAP29, STX3 and STX4 in FLAG-tagged FKBP51 co-IP (FLAG-IP) performed in SIMA-9 cells and whole cell extract (WCE) as control. Ab, antibody. **c)** ProteinSimple quantifications of secreted CTSD in vehicle, 300 nM Dex or 50 nM LLOMe treated SIMA-9 cells, co-treated with vehicle or Baf for 4 hours. Data shown as mean ± s.e.m. of n=4 biologically independent samples. Unpaired, two-tailed t-test was performed; *p < 0.05, **p < 0.01. Fc, fold change.

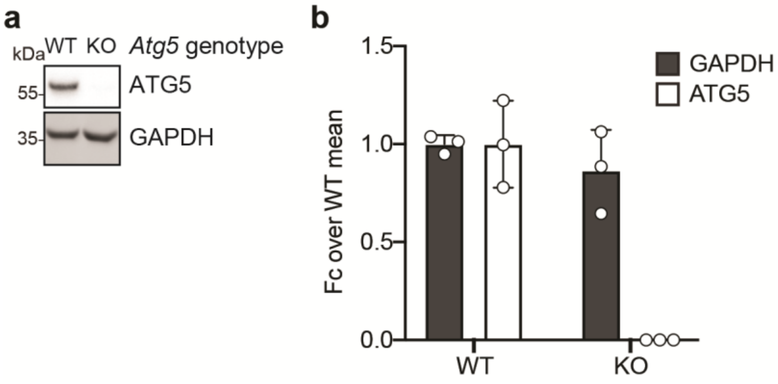

Figure S3

a) Western blotting for ATG5 and GAPDH in WT and *Atg5* KO SIM-A9 cells and **b)** relative quantifications. Data shown as mean ± s.e.m. of n=3 biologically independent samples.

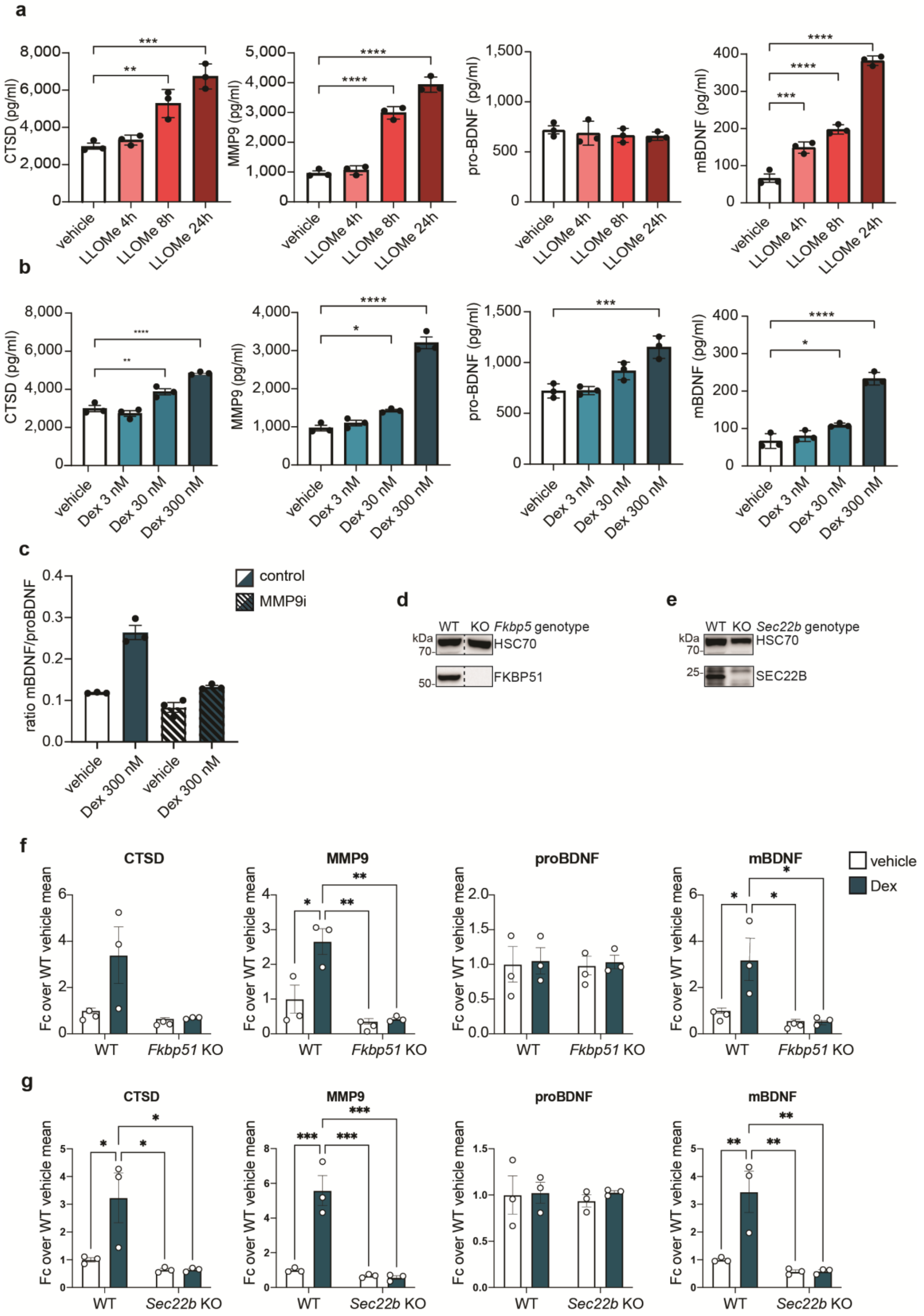

Figure S4

a) CTSD, MMP9**,** proBDNF, mBDNF levels from supernatants measured via ELISA after WT SIM-A9 cells were treated with 1 mM LLOMe for 4, 8 or 24 hours or vehicle or 24 hours; **b)** CTSD, MMP9**,** proBDNF, mBDNF levels from supernatants measured via ELISA after WT SIM-A9 cells were treated with 3 nM, 30 nM or 300 nM dexamethasone (Dex) or vehicle for 4 hours; **c)** mBDNF/proBDNF ratio from supernatants measured via ELISA after WT SIM-A9 cells were treated with 300 nM Dex, Dex + Mmp9 inhibitor I (MMP9i), or vehicle for 4 hours. a)-c) Data shown as mean ± s.e.m. of n=3 biologically independent samples. **d)** Western blotting for FKBP51 and Vinculin in WT and *Fkbp5* KO SIM-A9 cells. **e)** Western blotting for FKBP51 and Vinculin in WT and *Sec22b* KO SIM-A9 cells. The dotted line indicates a cut in the blot in order to close samples of interest that were separate on the blot. **f)** ProteinSimple quantifications of secreted CTSD, MMP9**,** proBDNF, mBDNF of WT and *Fkbp5* KO SIMA-9 cells treated with vehicle or 300 nM Dex for 4 hours. **g)** ProteinSimple quantifications of secreted CTSD, MMP9**,** proBDNF, mBDNF of WT and *Sec22b* KO SIMA-9 cells treated with vehicle or 300 nM Dex for 4 hours. f) and g) Data shown as mean ± s.e.m. of n=3 biologically independent samples. Tukey's multiple comparison tests were performed; only significant comparisons are shown. *p < 0.05; **p < 0.01; ***p < 0.001; ****p < 0.0001.

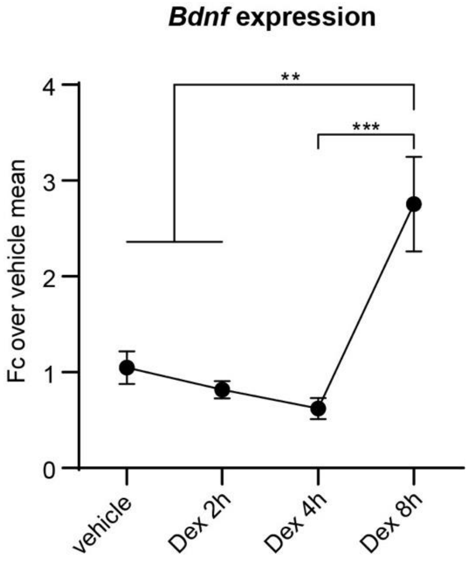

Figure S5

Quantitative polymerase chain reaction (qPCR) of *Bdnf* expressed in SIM-A9 cells treated with vehicle or 100 nM Dex for 2, 4 and 8 hours. Data shown as mean ± s.e.m. of n=3 biologically independent samples. Tukey's multiple comparison test was performed; **P < 0.01; ***P < 0.001.

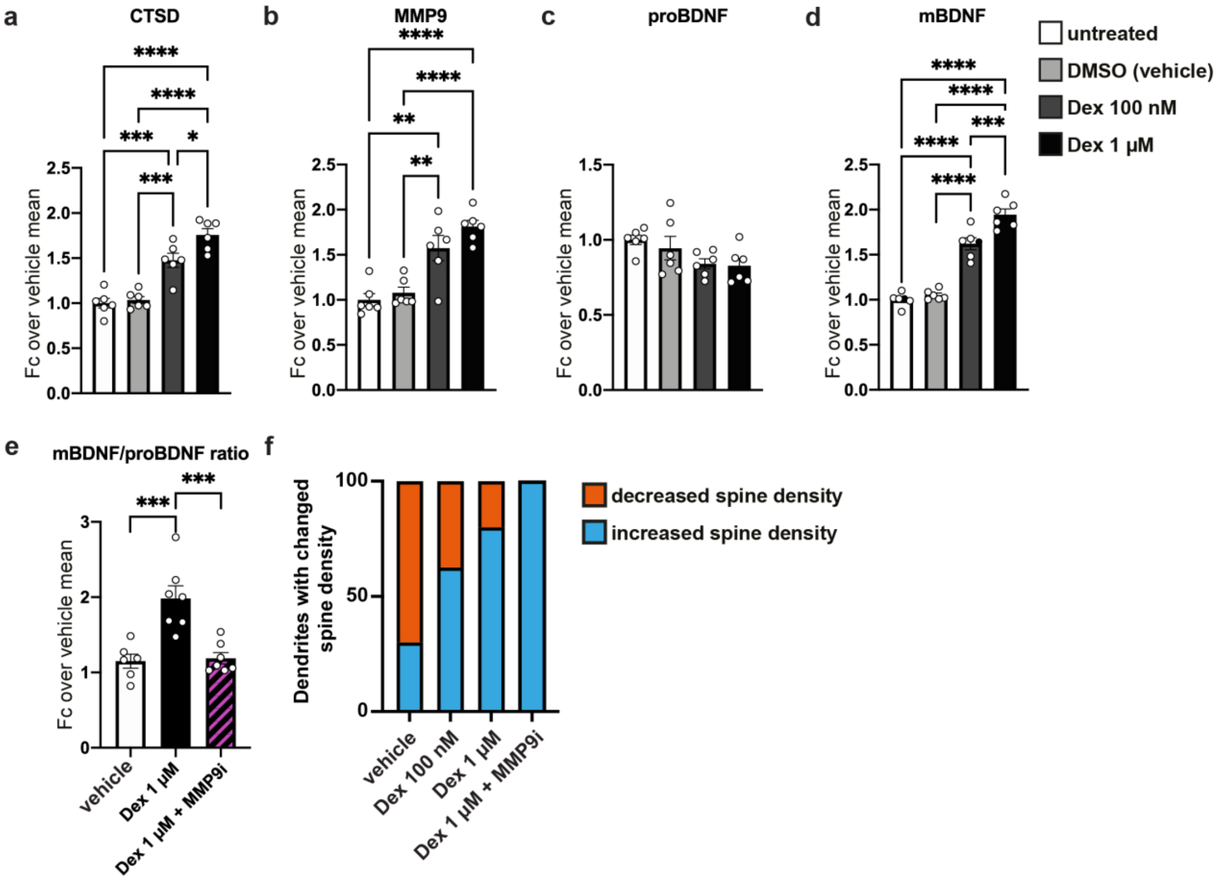

Figure S6

a-d) Quantifications of western blotting for CTSD, MMP9**,** proBDNF, mBDNF from culture media of OHSC treated with vehicle (DMSO), 100 nM Dex, 1 μM Dex or nothing (untreated) for 4-6 hours; Data shown as mean ± s.e.m. of n=6 biologically independent samples. **e)** Ratio of mBDNF over proBDNF relative to Fig. 6 d and e. Data shown as mean ± s.e.m. of n=7 biologically independent samples. Tukey's multiple comparison tests were performed; only significant comparisons are shown. *p < 0.05; **p < 0.01; ***p < 0.001; ****p < 0.0001. **f)** Quantification of imaged spine densities of OHSC treated with vehicle (DMSO), 100 nM Dex,

1 µM Dex and 1 µM Dex + MMP9i expressed as percentage of dendrites with changed spine densities over the 30 min imaging span, and subdivided into increased and decreased spine densities.

List of references to neuroplasticity-related proteins listed in Table 1

Protein	Reference
APP	[1–3]
JUN	[4,5]
VIM	[6,7]
CDC42	[8,9]
MMP9	[10–12]
CCL2	[13,14]
EEF2	[15–17]
HMGB1	[18]
IGF1	[19–21]
KDR	[22,23]
LRP1	[24–27]
PAK1	[28,29]
PAK3	[30,31]
STMN2	[32,33]
TF	[34]
TGFB1	[35–37]
CAT	[38,39]
HMOX1	[40]
JUNB	[41]

1. Deyts, C. *et al.* APP-Mediated Signaling Prevents Memory Decline in Alzheimer's Disease Mouse Model. *Cell Rep.* **27**, 1345-1355.e6 (2019).

2. Priller, C. *et al.* Synapse Formation and Function Is Modulated by the Amyloid Precursor Protein. *J. Neurosci.* **26**, 7212–7221 (2006).

3. Zheng, H. & Koo, E. H. The amyloid precursor protein: beyond amyloid. *Mol. Neurodegener.* **1**, 5 (2006).

4. Raivich, G. & Behrens, A. Role of the AP-1 transcription factor c-Jun in developing, adult and injured brain. *Prog. Neurobiol.* **78**, 347–363 (2006).

5. Alberini, C. M. Transcription factors in long-term memory and synaptic plasticity. *Physiol. Rev.* **89**, 121–145 (2009).

6. Adolf, A. *et al.* Release of astroglial vimentin by extracellular vesicles: Modulation of binding and internalization of C3 transferase in astrocytes and neurons. *Glia* **67**, 703–717 (2019).

7. Qian, B.-J. *et al.* Vimentin regulates neuroplasticity in transected spinal cord rats associated with micRNA138. *Mol. Neurobiol.* **51**, 437–447 (2015).

8. Kim, I. H., Wang, H., Soderling, S. H. & Yasuda, R. Loss of Cdc42 leads to defects in synaptic plasticity and remote memory recall. *eLife* **3**, (2014).

9. Imai, F. *et al.* Synapse Formation in Monosynaptic Sensory-Motor Connections Is Regulated by Presynaptic Rho GTPase Cdc42. *J. Neurosci.* **36**, 5724–5735 (2016).

10. Kaczmarek, L. MMP-9 in Control of Synaptic Plasticity: a Subjective Account. *Opera Medica Physiol.* **2**, 103–111 (2016).

11. Michaluk, P. *et al.* Influence of matrix metalloproteinase MMP-9 on dendritic spine morphology. *J. Cell Sci.* **124**, 3369–3380 (2011).

12. Lepeta, K. & Kaczmarek, L. Matrix Metalloproteinase-9 as a Novel Player in Synaptic Plasticity and Schizophrenia. *Schizophr. Bull.* **41**, 1003–1009 (2015).

13. Xie, R.-G. *et al.* Spinal CCL2 Promotes Central Sensitization, Long-Term Potentiation, and Inflammatory Pain via CCR2: Further Insights into Molecular, Synaptic, and Cellular Mechanisms. *Neurosci. Bull.* **34**, 13–21 (2018).

14. Zhou, Y., Tang, H., Liu, J., Dong, J. & Xiong, H. Chemokine CCL2 modulation of neuronal excitability and synaptic transmission in rat hippocampal slices. *J. Neurochem.* **116**, 406–414 (2011).

15. McCamphill, P. K., Farah, C. A., Anadolu, M. N., Hoque, S. & Sossin, W. S. Bidirectional regulation of eEF2 phosphorylation controls synaptic plasticity by decoding neuronal activity patterns. *J. Neurosci. Off. J. Soc. Neurosci.* **35**, 4403–4417 (2015).

16. Taha, E., Gildish, I., Gal-Ben-Ari, S. & Rosenblum, K. The role of eEF2 pathway in learning and synaptic plasticity. *Neurobiol. Learn. Mem.* **105**, 100–106 (2013).

17. McCamphill, P. K., Ferguson, L. & Sossin, W. S. A decrease in eukaryotic elongation factor 2 phosphorylation is required for local translation of sensorin and long-term facilitation in Aplysia. *J. Neurochem.* **142**, 246–259 (2017).

18. Tian, L., Rauvala, H. & Gahmberg, C. G. Neuronal regulation of immune responses in the central nervous system. *Trends Immunol.* **30**, 91–99 (2009).

19. LLorens-Martín, M., Torres-Alemán, I. & Trejo, J. L. Reviews: Mechanisms Mediating Brain Plasticity: IGF1 and Adult Hippocampal Neurogenesis. *The Neuroscientist* **15**, 134–148 (2009).

20. Ogundele, O. M., Pardo, J., Francis, J., Goya, R. G. & Lee, C. C. A Putative Mechanism of Age-Related Synaptic Dysfunction Based on the Impact of IGF-1 Receptor Signaling on Synaptic CaMKIIα Phosphorylation. *Front. Neuroanat.* **12**, (2018).

21. Liu, Z. *et al.* IGF1-Dependent Synaptic Plasticity of Mitral Cells in Olfactory Memory during Social Learning. *Neuron* **95**, 106-122.e5 (2017).

22. Cao, L. *et al.* VEGF links hippocampal activity with neurogenesis, learning and memory. *Nat. Genet.* **36**, 827–835 (2004).

23. De Rossi, P. *et al.* A critical role for VEGF and VEGFR2 in NMDA receptor synaptic function and fear-related behavior. *Mol. Psychiatry* **21**, 1768–1780 (2016).

24. Gan, M., Jiang, P., McLean, P., Kanekiyo, T. & Bu, G. Low-density lipoprotein receptor-related protein 1 (LRP1) regulates the stability and function of GluA1 α-amino-3-hydroxy-5-methyl-4-isoxazole propionic acid (AMPA) receptor in neurons. *PloS One* **9**, e113237 (2014).

25. May, P. *et al.* Neuronal LRP1 functionally associates with postsynaptic proteins and is required for normal motor function in mice. *Mol. Cell. Biol.* **24**, 8872–8883 (2004).

26. Maier, W. *et al.* LRP1 is critical for the surface distribution and internalization of the NR2B NMDA receptor subtype. *Mol. Neurodegener.* **8**, 25 (2013).

27. Pietrzik, C. U., Busse, T., Merriam, D. E., Weggen, S. & Koo, E. H. The cytoplasmic domain

of the LDL receptor-related protein regulates multiple steps in APP processing. *EMBO J.* **21**, 5691–5700 (2002).

28. Xia, S., Zhou, Z. & Jia, Z. PAK1 regulates inhibitory synaptic function via a novel mechanism mediated by endocannabinoids. *Small GTPases* **9**, 322–326 (2018).

29. Koth, A. P., Oliveira, B. R., Parfitt, G. M., Buonocore, J. de Q. & Barros, D. M. Participation of group I p21-activated kinases in neuroplasticity. *J. Physiol. Paris* **108**, 270–277 (2014).

30. Boda, B. *et al.* The Mental Retardation Protein PAK3 Contributes to Synapse Formation and Plasticity in Hippocampus. *J. Neurosci.* **24**, 10816–10825 (2004).

31. Meng, J., Meng, Y., Hanna, A., Janus, C. & Jia, Z. Abnormal long-lasting synaptic plasticity and cognition in mice lacking the mental retardation gene Pak3. *J. Neurosci. Off. J. Soc. Neurosci.* **25**, 6641–6650 (2005).

32. Peng, H., Derrick, B. E. & Martinez, J. L. Identification of upregulated SCG10 mRNA expression associated with late-phase long-term potentiation in the rat hippocampal Schaffer-CA1 pathway in vivo. *J. Neurosci. Off. J. Soc. Neurosci.* **23**, 6617–6626 (2003).

33. Riederer, B. M. *et al.* Regulation of microtubule dynamics by the neuronal growth-associated protein SCG10. *Proc. Natl. Acad. Sci. U. S. A.* **94**, 741–745 (1997).

34. Liu, K. *et al.* Transferrin Receptor Controls AMPA Receptor Trafficking Efficiency and Synaptic Plasticity. *Sci. Rep.* **6**, 21019 (2016).

35. Caraci, F. *et al.* A key role for TGF-β1 in hippocampal synaptic plasticity and memory. *Sci. Rep.* **5**, 11252 (2015).

36. Chin, J., Angers, A., Cleary, L. J., Eskin, A. & Byrne, J. H. Transforming growth factor beta1 alters synapsin distribution and modulates synaptic depression in Aplysia. *J. Neurosci. Off. J. Soc. Neurosci.* **22**, RC220 (2002).

37. Jaskova, K., Pavlovicova, M., Cagalinec, M., Lacinova, L. & Jurkovicova, D. TGFβ1 downregulates neurite outgrowth, expression of Ca2+ transporters, and mitochondrial dynamics of in vitro cerebellar granule cells. *Neuroreport* **25**, 340–346 (2014).

38. Ogłodek, E. A. Evaluation of ADMA, carbonyl groups, CAT and NKA in depressed patients with and without posttraumatic stress disorder. *Pharmacol. Rep. PR* **69**, 730–737 (2017).

39. Anaeigoudari, A. *et al.* Neuronal nitric oxide synthase has a role in the detrimental effects of lipopolysaccharide on spatial memory and synaptic plasticity in rats. *Pharmacol. Rep.* **68**, 243–249 (2016).

40. Schipper, H. M. & Song, W. A heme oxygenase-1 transducer model of degenerative and developmental brain disorders. *Int. J. Mol. Sci.* **16**, 5400–5419 (2015).

41. Hansson, A. C. & Fuxe, K. Time-course of immediate early gene expression in hippocampal subregions of adrenalectomized rats after acute corticosterone challenge. *Brain Res.* **1215**, 1–10 (2008).

42. Gassen, N. C. *et al.* Association of FKBP51 with Priming of Autophagy Pathways and Mediation of Antidepressant Treatment Response: Evidence in Cells, Mice, and Humans. *PLOS Med.* **11**, e1001755 (2014).
