# Supplementary material for: Stress-primed secretory autophagy promotes extracellular BDNF maturation by enhancing MMP9 secretion
Source: Nat Commun. 2021 Jul 30;12:4643. doi: 10.1038/s41467-021-24810-5 (PMC8324795; doi:10.1038/s41467-021-24810-5)
Supplement: Supplementary file 9 — Description of additional supplementary files [file 41467_2021_24810_MOESM9_ESM.docx]

Description of additional supplementary information

Title: supplementary table 1

Description: FKBP51 interactome

Title: supplementary table 2

Description: volcano plot results

Title: supplementary table 3

Description: reactome analysis results

Title: supplementary table 4

Description: SynGO results

Title: supplementary table 5

Description: literature mining neuroplasticity

Title: supplementary table 6

Description: gRNA oligos
