# Supplementary material for: Stress-primed secretory autophagy promotes extracellular BDNF maturation by enhancing MMP9 secretion
Source: Nat Commun. 2021 Jul 30;12:4643. doi: 10.1038/s41467-021-24810-5 (PMC8324795; doi:10.1038/s41467-021-24810-5)
Supplement: Supplementary file 10 — Reporting Summary [file 41467_2021_24810_MOESM10_ESM.pdf]

nature research

Corresponding author(s): Silvia Martinelli, Nils C. Gassen

Last updated by author(s): Jun 14, 2021

Reporting Summary

Nature Research wishes to improve the reproducibility of the work that we publish. This form provides structure for consistency and transparency in reporting. For further information on Nature Research policies, see Authors & Referees and the Editorial Policy Checklist.

Statistics

For all statistical analyses, confirm that the following items are present in the figure legend, table legend, main text, or Methods section.

n/a	Confirmed	
☐	☒	The exact sample size (*n*) for each experimental group/condition, given as a discrete number and unit of measurement
☐	☒	A statement on whether measurements were taken from distinct samples or whether the same sample was measured repeatedly
☐	☒	The statistical test(s) used AND whether they are one- or two-sided<br>*Only common tests should be described solely by name; describe more complex techniques in the Methods section.*
☒	☐	A description of all covariates tested
☐	☒	A description of any assumptions or corrections, such as tests of normality and adjustment for multiple comparisons
☒	☐	A full description of the statistical parameters including central tendency (e.g. means) or other basic estimates (e.g. regression coefficient) AND variation (e.g. standard deviation) or associated estimates of uncertainty (e.g. confidence intervals)
☒	☐	For null hypothesis testing, the test statistic (e.g. *F*, *t*, *r*) with confidence intervals, effect sizes, degrees of freedom and *P* value noted<br>*Give P values as exact values whenever suitable.*
☒	☐	For Bayesian analysis, information on the choice of priors and Markov chain Monte Carlo settings
☒	☐	For hierarchical and complex designs, identification of the appropriate level for tests and full reporting of outcomes
☒	☐	Estimates of effect sizes (e.g. Cohen's *d*, Pearson's *r*), indicating how they were calculated

Our web collection on statistics for biologists contains articles on many of the points above.

Software and code

Policy information about availability of computer code

Data collection	Scripts for automated literature search have been deposited on git hub in the following repository: https://github.com/silviamartinelli/paper-scripts.git
Data analysis	Proteomics data were processed and analyzed with MaxQuant (version 1.6.0.16) and Perseus software suite (v. 1.6.2.3). All other data were statistically analyzed with Prism version 8.1.1 (GraphPad Software, La Jolla California USA, www.graphpad.com). Gene onthology analyses were performed with Reactome (Pathway browser version 3.6; reactome database release 71, www.reactome.org) and SynGo version 1.1 (https://www.syngoportal.org/index.html)

For manuscripts utilizing custom algorithms or software that are central to the research but not yet described in published literature, software must be made available to editors/reviewers. We strongly encourage code deposition in a community repository (e.g. GitHub). See the Nature Research guidelines for submitting code & software for further information.

Data

Policy information about availability of data

All manuscripts must include a data availability statement. This statement should provide the following information, where applicable:

- Accession codes, unique identifiers, or web links for publicly available datasets
- A list of figures that have associated raw data
- A description of any restrictions on data availability

-The interactome mass spectrometry proteomics data have been deposited to the ProteomeXchange Consortium via the PRIDE39 partner repository with the dataset identifier PXD017328 and 10.6019/PXD017328. Reviewer account details: Username: reviewer67932@ebi.ac.uk. Password: y4kUvdEQ

-The secretome mass spectrometry proteomics data have been deposited to the ProteomeXchange Consortium via the PRIDE partner repository with the dataset identifier PXD017076. Reviewer account details: Username: reviewer53816@ebi.ac.uk. Password: GT6DRDWo.

Field-specific reporting

Please select the one below that is the best fit for your research. If you are not sure, read the appropriate sections before making your selection.

☒ Life sciences ☐ Behavioural & social sciences ☐ Ecological, evolutionary & environmental sciences

For a reference copy of the document with all sections, see nature.com/documents/nr-reporting-summary-flat.pdf

Life sciences study design

All studies must disclose on these points even when the disclosure is negative.

Sample size	No calculations were performed to pre-determine sample size. Where applicable, experiments were performed under the hypothesis that potential effect sizes would approximate those of previously examined, for which n=3-7 biological replicates were sufficient to characterize significant differences (Gassen et al., PLoS Med. 2014). When individual cells were analysed for counting ptGal3 positive lysosomes, about n=40 cells were analysed, again based on previous experience (Gassen et al., Nat. Comm. 2019) and in accordance with other published data (Maejima et al. EMBO J .2014). As the effect size of the secretomics experiments was unknown prior to this work, we pre-determined they should be performed in biological triplicates, to enhance sensitivity to potentially small effect sizes and better ensure that the results would be suitable for subsequent multivariate analysis. Our mice-microdialysate data reflect the sample sizes (n=4) from a previous study (Anderzhanova et al. Neurobiol of Stress. 2020).
Data exclusions	No data were excluded
Replication	The cell experiments were performed in at least three biological replicates. All attempts at replication were successful.
Randomization	No randomization was necessary as n was low enough to allow to conduct the experiments on all the samples (for each experiment) in parallel.
Blinding	In tfGal3 assay, the count of fluorescent puncta was performed manually in a blinded way. All other analyses were performed automatically and had therefore no necessity for blinding.

Reporting for specific materials, systems and methods

We require information from authors about some types of materials, experimental systems and methods used in many studies. Here, indicate whether each material, system or method listed is relevant to your study. If you are not sure if a list item applies to your research, read the appropriate section before selecting a response.

Materials & experimental systems

n/a	Involved in the study	
☐	☒	Antibodies
☐	☒	Eukaryotic cell lines
☒	☐	Palaeontology
☐	☒	Animals and other organisms
☒	☐	Human research participants
☒	☐	Clinical data

Methods

n/a	Involved in the study	
☒	☐	ChIP-seq
☒	☐	Flow cytometry
☒	☐	MRI-based neuroimaging

Antibodies

Antibodies used	Primary antibodies used: LC3B-II/I (1:1000, Cell Signaling, #2775), FLAG (1:7000, Rockland, 600-401-383), FKBP51 (1:1000, Bethyl, A301-430A), FKBP51 (1:1000, Cell Signaling, #12210), ACTIN (1:5000, Santa Cruz Biotechnology, sc-1616), GAPDH (1:8000, Millipore CB1001), TRIM16 (1:1'000, Bethyl A301-160A), CTSD (for Human) (1:50, Abcam, ab6313), CTSD (for Mouse) (1:50, Abcam, ab207549), SNAP29 (1:1000, Sigma, SAB1408650), SNAP23 (1:1000, Sigma, SAB2102251), STX3 (1:1000, Sigma, SAB2701366), STX4 (1:1000, Cell Signalling. #67657), GAL8 (1:1'000, Santa Cruz, sc-28254), GAL3 (1:1'000, Santa Cruz, sc-32790), SEC22B (1:1000, Abcam, ab181076), Secondary antibodies: anti-rabbit IgG, HRP-linked antibody (1:10,000, Cell Signalling Technology, #7074), anti-mouse IgG, HRP-linked antibody (1:10,000, Cell Signalling Technology, #7076)
Validation	The antibodies were validated by the manufacturer. Primary antibodies: LC3B #2775 (validated for WB; species reactivity H M R); FLAG 600-401-383 (validated for WB IF ELISA; highly specific); FKBP51 A301-430A (validated for WB IP IF; species reactivity H); FKBP51 #12210 (validated for WB IP; species reactivity H M R Mk); ACTIN sc-1616 (validated for WB; species reactivity H M R); GAPDH CB1001 (validated for WB IF ELISA; species reactivity H M R Mk Canine Chicken Fish Porcine Frog Rb); TRIM16 A301-160A (validated for WB; species reactivity H M); CTSD ab6313 (validated for WB; species reactivity H); CTSD ab207549 (validated for WB IP IF; species reactivity H M); SNAP29 SAB1408650 (validated for WB; species reactivity H R); SNAP23 SAB2102251 (validated for WB; species reactivity H); STX3 SAB2701366 (validated for WB IF; species reactivity H M R); STX4 #67657 (validated for WB IP IF; species reactivity H M); GAL8 sc-28254 (validated for WB IF; species reactivity H M R); GAL3 sc-32790 (validated for WB IP IF; species reactivity H M R); SEC22B ab181076 (validated for WB IP IF; species reactivity H M R).

Secondary antibodies were also evaluated for cross-reactivity by the manufacturer and used consistently with the manufacturer's recommendations.

Eukaryotic cell lines

Policy information about cell lines

Cell line source(s)	SH-SY5Y line from Sigma-Aldrich, #94030304-1VL (https://www.sigmaaldrich.com/catalog/product/sigma/cb_94030304?lang=de&region=DE)<br>SIM-A9 line from Kerafast, #END001 (https://www.kerafast.com/item/985/microglial-cell-line-sim-a9)<br>HEK 293 (ATCC CRL-1573)
Authentication	Commercial cell lines were not further authenticated in the lab
Mycoplasma contamination	All cell lines tested negative for mycoplasma contamination
Commonly misidentified lines (See ICLAC register)	No commonly misidentified cell lines were used in the study.

Animals and other organisms

Policy information about studies involving animals; ARRIVE guidelines recommended for reporting animal research

Laboratory animals	C57BL/6 male mice (Martinsried, Germany), FKBP51-KO and respective WT males, Thy1-GFP-M (Jackson Laboratory Stock #007788) the gender was not determined from pubs
Wild animals	This study did not involve wild animals
Field-collected samples	This study did not involve field-collected samples
Ethics oversight	All procedures were done in accordance with European Communities Council Directive 2010/63/EU and approved by Government of Upper Bavaria.

Note that full information on the approval of the study protocol must also be provided in the manuscript.
